# Successful School Interventions for Students with Disability During Covid-19: Empirical Evidence from Australia

**DOI:** 10.1007/s40299-022-00659-0

**Published:** 2022-04-09

**Authors:** Catherine Smith, Massimiliano Tani, Sophie Yates, Helen Dickinson

**Affiliations:** 1 Melbourne Graduate School of Education, University of Melbourne, Melbourne, VIC Australia; 2 School of Business, University of New South Wales, Canberra, ACT Australia

**Keywords:** Covid-19, Remote learning, School closures, School interventions, Social supports, Students with disability

## Abstract

Children and young people with disability are a “vulnerable” population within a pandemic context as they face structural inequities and discrimination as a result of their impairments. In this paper, we report research that sought to examine the learning experiences of children and young people with disability during the COVID-19 pandemic. We wanted to understand how this group fared and whether different interventions impacted on these experiences. Data were collected from an online survey organized by Children and Young People with Disability Australia (CYDA) that garnered responses from more than 700 families. The study contributes empirical evidence to the growing literature about COVID-19-related impacts on learners already recognized as experiencing multiple disadvantages in schooling. We find some significant gaps in supports offered to students with disability and their families. Notwithstanding that some students did not receive any support from their schools, where supports were offered, social supports had the greatest positive impact on feelings of learner engagement. Our findings support key propositions in the social and emotional learning literature, namely that particular resourcing should be dedicated to social interaction and feelings of belonging as these are crucial to learners engaging in learning processes. There are clear implications of these findings in terms of what educational institutions might do to help engage students with disability in remote learning.

**Supplementary Information:**

The online version contains supplementary material available at 10.1007/s40299-022-00659-0.

## Introduction

The COVID-19 pandemic has disrupted the learning activities of many children and young adults with and without disability in various countries around the world. While all children and young people faced challenges during remote learning, there is good reason to suspect those with disability fare worse during this period. When schools a shut, a range of concerns continue to be raised regarding the negative repercussions for children from ‘vulnerable’ backgrounds such as children in low-income families and children with disability (Brown et al., 2020; Drane et al., 2021; Yates et al., 2021). Changes in routine, lack of experience with technology and digital learning, parental overwhelm and increased social isolation are examples of the issues young people with disability and their families face (Page et al., 2021). Even pre-pandemic, children and young people with disability faced significant inequities in accessing education and on average had poorer educational outcomes than their non-disabled counterparts (Anderson & Boyle, 2019).

This paper explores the experiences of children and young people with disability and their families over the first remote learning period Australia experienced in the COVID-19 pandemic. Our research aimed to explore:

- How children and young people with disability and their families had their education disrupted;
- What the impact of this was in terms of engagement with learning, learning supports, mental health and feelings of isolation; and,
- What supports had been offered.

This paper reports findings from over 700 respondents to an online survey conducted by Children and Young People with Disability Australia (CYDA), the national peak body that represents children and young people (aged 0–25) with disability. The survey revealed that most children and young people with disability felt forgotten in the transition to remote learning. Many of their usual supports dropped away and educational institutions failed to make appropriate accommodations to engage in learning. Some did, however, receive extra or special educational materials, supervision, specific aides, online care services, and social support. This paper discusses the effectiveness of these interventions for the learning experiences of children and young adults with disability, and whether all types of support were equally helpful or some were more highly associated with better outcomes. These findings provide valuable information for reviewing the provision of effective inclusive education.

The paper finds that of all types of support offered, social supports had the greatest association with better outcomes in learner engagement. While educational interventions seemed to make some difference, social supports were more highly associated with good engagement outcomes. This finding supports a key theme in the social and emotional learning literature that socialization is a fundamental conduit to foster learning among students with disability, and that activities catering for it ought to feature prominently among the set of supports being provided by school and government.

The argument in this paper is structured as follows. After briefly reviewing the relevant literature, we present the data and the methodology applied. We then present the results and draw some concluding remarks.

## Background

In the early stages of the COVID-19 pandemic, Australian health authorities and state and federal governments responded to the propagation of the infection by recommending people reduce their face-to-face interactions. This quickly turned into mandatory lockdowns of economic activities and school closures after the first few deaths. School shutdowns were implemented in all states by late March 2020 and remained closed in most states until May (Moloney & Moloney, 2020). School holidays were brought forward and extended while remote learning platforms were adopted. Although some schools remained opened for some children, including some special schools and classes for the children of workers considered ‘essential’ during the pandemic (e.g., nurses, doctors and some transport and shop workers), the disruption to schooling (and parents), was widespread. Furthermore, the transformation of schooling into a set of learning activities that predominantly took place online had dramatic consequences for households’ wellbeing: parents, often ‘temporarily suspended’ from work or even laid off due the sharp downward trend in economic activity, suddenly found themselves juggling the demands of finding or maintaining an income and those of parenting children engaged in remote learning. The OECD (2020) issued a report about remote learning experiences identifying a critical gap in communication between teachers and parents/carers across the globe and advised that effective communication between school and home is the ‘critical element’ (p. 8) in remote learning. Unsurprisingly, parents reported rapidly worsening mental health conditions as a product of these significant pressures (Cheng et al., 2021). Lockdowns and restricted access to on-campus learning continues to be the lived experience of many Australian young people. This paper reports on data from the first stages of the pandemic in Australia and the perceived impacts of this initial wave of policy responses.

It is well established in the literature that children and young people with disability face significant challenges with education. Besides academic challenges, these at times include teachers’ preparedness to deal with students with disability (Mason & Hedin, 2011; Richards et al., 2007), a high likelihood of being bullied (Bourke & Burgman, 2010; Carter & Spencer, 2006; Rose & Espelage, 2012), and experiences of exclusion and rejection (Davis & Watson, 2001; Krull et al., 2014; Russell, 2003). As an example, although inclusion in regular classrooms may allow students who are neuro-diverse to be involved in the social structure of their classroom, they still report loneliness, poorer friendship quality and social network status compared with their classmates (Locke et al., 2010).

Teachers and school personnel can help to overcome the difficulties experienced by children and young people with disability when they are enabled to provide a learning environment that is both supportive and inclusive. There is good evidence to show intensive and explicit social and social-emotional skill development can disrupt patterns of bullying (Espelage et al., 2015; Rose & Espelage, 2012); build empathy, understanding, connection for and with students with and without disability through restorative justice practices (Hulvershorn & Mulholland, 2018); and help students with learning disabilities as well as other students develop coping skills to counter anxiety (Khodadadi et al., 2017). Children with disability can and do thrive when they develop good relationships with teachers and strong bonds with the school (Murray & Greenberg, 2001). These interventions do not have a negative impact on children without disability, who are unaffected in their academic achievements when they learn in inclusive classrooms. This experience can also reduce prejudice towards people with disability and shape children and young people to be more accepting and supportive of people who are different from themselves (Molina Roldán et al., 2021). For example, the social effects of supported inclusion include reduction of fear, hostility, prejudice, and discrimination as well as increase of care, acceptance, and understanding (Kart & Kart, 2021).

Social activity and support are fundamentally important for learning among children and young adults with disabilities (Campbell & Gilmore, 2014; Gilmore et al., 2016), as highlighted by the educational psychology literature in studies carried out around the world (Adair et al., 2015; Cavioni et al., 2017), including in Australia (Foley et al., 2012; Raghavendra et al., 2015). Social supports are broadly categorized as meeting needs in an emotional, tangible, or informational way (Schaefer et al., 1981; Tandon et al., 2013). Young people with disability report their wellbeing relies on feeling supported, respected, and capable to make self-determining choices as individuals (Colon-Cabrera et al., 2021). The perception that supports are available is a strong protective factor for wellbeing (Haber et al., 2007; Harandi et al., 2017). Social support has long been evidenced as protecting against stress, and gives people the feeling of being loved, cared for and respected, as well as a sense of belonging to a network (Cobb, 1976). While parent relationships are seen as the strongest protective factor for wellbeing and mental health, social supports through peers provide behavioral and emotional support and supportive teacher relationships are most likely to help students remain engaged with schooling (Campbell & Gilmore, 2014). These relationships are not created instantly but are built and reinforced over time. These relationships are particularly important in helping teachers to understand the needs of students who are neuro-diverse or have disability (Hood, 2020).

The arrival of COVID-19 in Australia forced social distancing rules that included the closure of schools and the transfer of teaching and learning to an online setting. The ensuing disruption was enormous for students and their families, as not only did they have to rapidly adjust to new teaching and learning systems, but also to endure the resulting lack of direct contact with peers, teachers, and support staff. While the appropriateness of school closures as a response to the pandemic has been debated (e.g., Leask & Hooker, 2020), there is little doubt the closures had negative short-term effects for vulnerable students, and likely long-term effects that will manifest in future (Drane et al., 2021; Fitzgerald et al., 2020). Twitter data analysis by Gleason et al. (2020) indicates that among the barriers faced by people with disability during the COVID-19 crisis, the rapid transition to remote online learning exacerbated accessibility issues. In this paper we explore the experiences of children and young people with disability and their families and what appropriate adjustments were made for this group to guard against negative impacts.

## Methodology

At the outset of the pandemic, CYDA surveyed nearly 700 families across Australia to capture the impact of the COVID-19 on children and young people with disability and their families, in order to understand the implications of the pandemic and the issues it was raising for this group (Dickinson et al., 2020). Uncertainty about access to education services was raised as a particular issue so a second online survey was devised to explore issues related to education specifically. We report on these data in this paper.

The online survey (hosted on Survey Monkey) was active for just under seven weeks, opening on the 28th of April 2020 and closing on the 14th of June. The survey contained a combination of questions on perceptions of educational outcomes to which respondents had to agree or disagree using a Likert score (1 = strongly disagree; 5 = strongly agree), as well as free text questions focusing on the impact of COVID-19 on access to education and engagement in learning and the community.

We downloaded the results into Excel and applied regression analysis to study the possible relationships between multifaceted perceptions of learning outcomes experienced by students during the pandemic We did this to identify possible influencing factors, controlling for demographic, locational, and educational characteristics of each student with disability represented in the study (for details see the Technical Appendix). In particular, we were interested in:

- whether or not the various types of support provided during the pandemic contributed to sustain student learning (at least as perceived by the family member or the student responding to the survey) as well as reducing feelings of loneliness and isolation, and
- identifying which support types (if any) were most associated with better learning outcomes such as engagement in learning or reduced perceptions of students’ loneliness and isolation.

In doing so we drew on answers provided to four Likert-scaled questions posed in the survey about perceptions of learning experiences, which we refer to as ‘outcomes’—namely:

- The student receives adequate support in their education
- The student is made to feel part of the learning community
- The student is engaged in his/her learning
- The student feels more socially isolated from his/her peers

In another question we asked whether there had been any other impacts of COVID-19 felt outside of education. One option was to indicate a decline in mental health and wellbeing (e.g. anxiety, fear or stress). We used the response to this question as a factor that might influence the four possible outcomes reported above, adding it to the list of explanatory variables.

The explanatory variables also included the age, gender, cultural background, and location of the respondents as well as their educational arrangements (type of school, attendance, NDIS eligibility, existence of an Individual Education/Learning Plan [IEP]), and an index of the impact of COVID-19 on the family, based on self-reported information (e.g. loss of income or job, access to food supply).

The regression analysis performed is based an Ordinary Least Squares (OLS) applied to the statistical model:

$${y}_{i}=\alpha +{X}_{i}\beta +\gamma {Z}_{i}+{\varepsilon }_{i}$$

where:

- ${y}_{i}$ is the educational outcome of interest experienced by student *i*. For example, in the case of whether the student receives adequate support in their education, the variable ${y}_{i}$ includes the values reported in the survey: namely, 5 if the survey respondent strongly agrees with the statement, 4 if s/he agrees, 3 for a neutral answer, 2 for disagreeing and 1 if the respondent strongly disagrees with the statement. A similar approach is used for each of the remaining outcomes summarized in 2–4 above, and run separate regressions for each of the four possible educational outcomes, generating four sets of results.
- ${X}_{i}$ is a set of independent variables that control for gender, age group, support received before the pandemic, whether studying full-time, mental health status, non-English-speaking background, whether aboriginal or Torres Strait islander, if funded by NDIS, whether has individual education program in place, type of school, and location (urban and in which state);
- ${Z}_{i}$ is the key explanatory variable, namely a set of answers about the support received during the pandemic. This set contains five components: namely, whether the support took the form of curriculum support and a support worker, specific aides and equipment, supervision, social support, and care services (assistance with personal care + behavioral support + access to specialist allied health).

We apply vector ${Z}_{i}$ in two alternative specifications: first, as a 3-category variable, which we label “version A”, with values of 0 if no support was received, 1 if only one type of support was received; and 2 if two or more types of support were received. In the second specification, we use the five components of vector ${Z}_{i}$ independently, as five separate indicators (“version B”).

## Findings

The survey was distributed online to members of CYDA (more than 5000 people), and 719 respondents completed it: of these, 95% were family members of a child or young adult with disability, while 5% were young adults with disability. We control for the self- or proxy-reported nature of their answers with an ad hoc dummy variable in the empirical analysis finding mixed evidence about its significance in the various specifications (see Tables A1 and A2 in the Appendix).

In almost two thirds of cases, mostly in the largest urban centers, respondents indicated that students faced a shift in the learning environment from face-to-face to online, while in another 15% of cases school closures were experienced. While many students with disability received substantive support from their education facility before the onset of COVID-19, many of the supports were not carried over into the pandemic. This was particularly notable in relation to the provision of social support and education, as school learning support workers (who would usually support the student to engage in the classroom) were not permitted to enter students’ homes.

Only half of the respondents reported that schools provided extra curriculum and learning materials to their children during the pandemic and just under half (46%) indicated that contact with the education provider was regular, ensuring accessibility and continuity of learning.

Table 1 reports the mean and standard deviation of the key variables used in the analysis. As shown in the bottom row, the number of respondents reduces to 618 (from the initial total of 719) due to missing answers to questions about educational outcomes—the main dependent variables in the analysis.

**Table 1:** Descriptive statistics

Variables	Mean	Standard deviation
Dependent variables (based on 5-point Likert scale)		
The student receives adequate support	2.43	1.22
The student is made to feel part of the learning community	2.79	1.27
The student is engaged in his/her learning	2.65	1.29
The student feels more socially isolated	3.99	1.12
Key independent variables		
Index of support *during* COVID		
Version A (range 0–8)	1.22	1.65
Version B		
Education support (curriculum modification + individual support worker) (range 0–2)	0.45	0.50
Specific aides and equipment (range 0–1)	0.11	0.32
Supervision (range 0–1)	0.12	0.30
Social support (range 0–1)	0.10	0.30
Care services (assistance with personal care + behavioral support + access to specialist allied health) (range 0–3)	0.22	0.42
Other independent variables		
Index of COVID impact (range 0–12)	2.74	2.18
Index of support *before* COVID (range 0–8)	3.82	2.68
Respondent is a young adult with disability^a^	0.04	0.19
Mental health	0.61	0.49
Age group	2.47	2.12
Gender is female^a^	0.33	0.47
Non-English speaking background^a^	0.05	0.23
Aboriginal or Strait Island heritage^a^	0.04	0.19
Full-time student^a^	0.90	0.31
Extra funds received^a^	0.57	0.50
Independent education plan is in place^a^	0.71	0.45
National disability insurance scheme recipient^a^	0.74	0.44
Located in urban/metropolitan area^a^	0.65	0.48
School type		
Non-government^a^	0.20	0.40
Other^a^	0.12	0.33
Special school^a^	1.10	.468
State		
VIC^a^	0.32	0.47
QLD^a^	0.24	0.43
Other^a^	0.19	0.39
Number of observations	618	

^a^Proportion of affirmative responses—i.e. range 0–1

Students with disability represented in the survey typically attended school or education on a full-time basis (90%) and were enrolled in Australia’s National Disability Insurance Scheme (74%—*N* = 455), indicating a relatively high level of support needs. In 71% of cases (*N* = 441) students had an Individual Learning Plan or Individual Education Plan in place as a pathway to learning. Most respondents had an English-speaking background (95%), and few had an Indigenous background (4%), although this is proportional with the Indigenous population nationally.

Students attended predominantly government schools (68%) and, less commonly, non-government and other (e.g. religious) schools. Most of the schools were in metropolitan/urban centers but well spread around the three states supplying the bulk of responses (Victoria, NSW, and Queensland).

The responses also highlighted that not every student covered by the CYDA survey had been negatively affected by COVID-19 (2.79/5), and most had received school support before the outset of the pandemic (3.89/5). The most common form of support received during the pandemic was educational (45%), followed by care services (22%) and specific equipment, supervision, and social support (about 10% in each case).

The key results of the regression analyses using version A and B are reported in Tables 2 and 3, respectively, while the corresponding full set of estimates obtained in each case are reported in Tables A1 and A2 in the Technical Appendix. In each Table, the outcomes are reported along the columns, while the explanatory variable(s) are reported in the rows.

**Table 2:** Regression baseline results: educational outcomes

Dependent variable controls	The student receives adequate support	The student is made to feel part of the learning community	The student is engaged in his/her learning	The student feels more socially isolated
Version A; 1 type of intervention only	.358*** (.109)	.240** (.112)	.105 (.117)	− .105 (.110)
2 + types of intervention	1.09*** (.115)	.881*** (.120)	.474*** (.130)	− .182* (.106)
Mental health	− .412*** (.094)	− .297*** (.099)	− .487*** (.107)	.524*** (.093)
*N*	618	616	615	616

*Notes* Standard error in parentheses. Point estimates different from zero at 10%, 5%, and 1% level of statistical significance are starred with *, **, and ***. Coefficients that are statistically no different from zero are presented in the full set of estimates in Table A1 in the Appendix

**Table 3:** Regression extension on what support worked: educational outcomes

Dependent variable controls	The student receives adequate support	The student is made to feel part of the learning community	The student is engaged in his/her learning	The student feels more socially isolated
Index support during COVID (version B)				
Education support	.447*** (.096)	.291*** (.100)	.074 (.106)	− .009 (.093)
Specific aides and equipment	.489*** (.164)	.328** (.154)	.108 (.172)	− .257* (.140)
Supervision	.380** (.164)	.421** (.164)	.315* (.180)	.104 (.138)
Social support	.525*** (.189)	.440** (.182)	.575*** (.197)	− .308** (.160)
Care services	.163 (.136)	.172 (.128)	.029 (.143)	− .107 (.119)
Mental health	− .408*** (.094)	− .287*** (.098)	− .469*** (.106)	.512*** (.093)
*N*	618	616	615	616

*Notes* Standard error in parentheses. Point estimates different from zero at 10%, 5%, and 1% level of statistical significance are starred with *, **, and ***. Coefficients that are statistically no different from zero are presented in the full set of estimates in Table A2 in the Appendix

Notwithstanding that a significant proportion of students with disability did not receive any form of support, the estimates reported in Table 2 suggest those who did benefited significantly from it. In particular, the support received was correlated with maintaining their learning engagement and reducing feelings of social isolation. Specifically, those who received only one type of support reported on average a 24% improvement in feeling part of their learning community relative to not receiving support, and a 35.8% improvement on the question of whether the student receives adequate support in their education. These increases are large in magnitude, and are statistically significantly different from zero at the 1% level: in other words, there is a less than 1% chance that the effect is zero. In contrast, no detectable effects were found for engagement (student is engaged in their learning: + 10% but the difference is statistically not significant), and the feeling of social isolation (− 10.5% but again not statistically significant).

In addition, those who received two or more types of support (about 30% of respondents) experienced very large and statistically significant improvements relative to no support: on average they felt an 88% improvement on the score measuring whether the student is made feel part of their learning community, and a 109% increase on the score measuring whether the student receives adequate support in their education. At the same time, relative to those receiving no support, these respondents reported a 47.5% increase on perceiving the student to be engaged in their learning, and a decline of 18.2% on whether the student feels lonely. These results suggest that support was effective relative to no support, and that it was most effective when it was more intense—i.e. when more than one type of support was provided.

When the effectiveness of each type of support was measured individually, we found that social support had the strongest association with improved educational outcomes, as it was strongly related to each of the possible outcomes across the columns of Table 3. Other types of support were associated with some positive outcomes, but not across the full range of perceived learning outcomes. Educational support and extra aides and equipment were associated with improved perceptions that the student was adequately supported by the school, and was made to feel part of the learning community. However, this type of support had no detectable effect on whether the student was engaged in learning activities, or whether they felt more socially isolated. Additional supervision was positively associated with feeling supported and part of the learning community, as well as feeling engaged in learning, but had no association with loneliness and feeling socially isolated. Care services may be helpful but their effect was practically nil.

Overall, the estimates in Table 3 point to a single element that stands out across the various types of support that schools have provided: social support. This is a large category, but would typically help to connect children and young people to their peers in meaningful ways. Social support was significantly associated with better learning processes and reducing isolation of students with disability. The impact of social supports was much more significant than even education supports. However, social supports were among the support types (along with support workers) hit hardest, with far fewer of these being provided during the pandemic than before. Support from teachers was identified as sporadic by some of our participants in free text comments, with some reporting having no contact at all. The importance of strong relationships between students and teachers in school engagement was evident across many of the participants’ responses.

In identifying the things that worked, participants named quite different requirements, depending on the different functional needs of the young person. Many carers and young people identified that the lack of communication and connection left them feeling forgotten and isolated. When identifying what ameliorated this, the main social supports identified were consistent but not too frequent contact, the opportunity to connect with peers, having school work that was the same content as peers but modified appropriately and knowing there was somewhere to go to for help and someone who cared to check in or respond to questions and concerns.

## Discussion

Social support is a protective factor for mental health and wellbeing of young people with intellectual disability (Campbell & Gilmore, 2014) and we assert from our findings that it is a key consideration when supporting the learning of young people in remote teaching and learning. Our results suggest that social distancing, school closures and learning online have disrupted the educational lives of these students and their families. To mitigate these disruptions, social supports are the form of support that are most valued by the students with disability and families participating in this study: the coefficient for this component is both large and always significantly different from zero across the three positive and one negative learning outcomes used as dependent variables. We argue that learning and engagement that take place via social activities and with social support are most likely to have the most relevant effect on learning and engagement during this prolonged period of disruption.

Children and young people with disability, already coping with discrimination and social exclusion before the pandemic, felt and are feeling the impact of COVID-19 quite severely, especially when schools had to close and online learning activities were often the only option left to continue schooling (Page et al., 2021). As the crisis has continued, and chronic uncertainty, multiple disruptions, remote learning and social isolation has been the experience of many young people, the adverse effects on mental health are likely to become more prevalent (Xiong et al., 2020). Being cut off from peers and teachers removed a fundamental channel through which children and young adults with disability grow as students and individuals. The concerns expressed by our participants about being invisible and undervalued as members of their communities are identified more broadly in research identifying structural inequalities within many of the services and resources for people with disability (Colon-Cabrera et al., 2021). Attention to providing social support and opportunities for social activity with social-emotional support and instruction during synchronous online instruction can provide inclusion opportunities (following Kart & Kart, 2021). These supports can include developing supported social-emotional skills to seek help in coping with anxiety (following Khodadadi et al., 2017), addressing some of the social isolation by securing ongoing and further relationships with teachers and peers. Disruptions to the routines of social activity and support that help young people, especially young people with disability thrive (Gilmore et al., 2016) can be further addressed with social supports. They promote self-efficacy and self-determining choices (Colon-Cabrera et al., 2021) reinforcing the communication of support needs for academic support and improving feedback loops between educators and students (Campbell & Gilmore, 2014; Hood, 2020).

The result that social support was the single most important form of support positively associated with all aspects of perceived learning outcomes covered in the CYDA survey, while reducing feelings of isolation, is a clear indication of what schools could do to engage children and young adults with disability in remote learning, and where to direct financial resources. Drawing on the strategies identified in our textual data and the literature on social supports for disability, scaffolds and supports for peer relationships between all students will benefit all students. Interactive activities, such as collaborative learning activities online, opportunities to participate in social-emotional learning in group communication and collaboration were among those strategies identified as effective. Explicit attention to skills in building and maintaining relationships are likely to support and maintain social networks (Page et al., 2021; Drane et al., 2021) can help to develop coping skills and opportunities for peer to peer and teacher to student understanding (Cavioni et al., 2017). Connecting the experiences of learning to home and taking time to assess the challenges and strategies that students used to cope and engage with their learning in different environments will assist in skill and empathy building (following Espelage et al., 2015; Hulvershorn & Mulholland, 2018; Khodadadi et al., 2017; Masi et al., 2021). Connecting with young people and their families to inform schools and teachers about what works, what might work better, and what has been learned from the experiences of remote learning, particularly around social support, will further allow this work to be done with dignity, informed by the knowledge and understanding of the young person’s experience (Children and Young People with Disability [CYDA], 2020; Colon-Cabrera et al., 2021).

The importance of social support may be also at the core of why non-government schools seem to have been more effective in their support relative to government schools, in that they may cater for a more homogeneous group of students, whose needs were easier to organize. It is also possible that non-government schools have more resources to support students with disability, or, alternatively, that families of students with disability attending non-government schools are richer or better resourced to support their children’s education (Vaz et al., 2015).

Turning to the insights from families and young people in the data, building skills in the use of technology and computers including how to log in, manage apps and practice communication in different digital mediums for young people needs more attention, as does the communication around support strategies between school and home (Long et al., 2021). Importantly, attention to professional development for teachers preparing them to support and inform the learning of young people with disability in digital and in-person teaching, drawing on the experiences of isolation reported during remote learning, would further support progress in providing inclusive learning experiences.

Finally, this survey was not without its limitations. As we have noted, the vast majority of responses came from family members so one of the gaps in this dataset is the voices of the children and young people with disability. The survey was distributed and promoted by CYDA and social media and may not therefore be a representative sample. The survey was only open for a limited amount of time and was restricted to an online platform so those lacking access to the internet would not have been able to participate.

## Concluding Remarks

The results suggest that receiving support during COVID-19 made a substantive and positive contribution to maintaining learning engagement with classmates and school, and reducing feelings of isolation. When the type of intervention is disaggregated, the component that overwhelmingly emerges as being most significant in generating these results is social supports. Maintaining contact with the student has been the most valuable type of intervention for those affected by school lockdowns, ahead of receiving equipment, supervision, and other care services. This is perhaps not surprising, as COVID-19 most directly hit children and young adults with disability via the social separation imposed by social distancing.

Notwithstanding that a substantial proportion of students with disability did not receive any support, the results suggests a wide range of potential changes that might be made to better protect children and young people and their families from experiencing similar sorts of issues in the face of another wave of infection or other disaster scenarios. It is evident that receiving some support has an impact on engagement in learning communities, learning itself, and reduction in social isolation. Further, two or more supports had a significant and substantial positive association with good outcomes, over and above one intervention. This suggests that where children received careful and planned responses, this mitigated against negative impacts and improved learning engagement. It is an important observation as it suggests that actions by schools do have valuable impacts for children and their families. Within support types, social support provisions seem to have the greatest positive association. This intuitively makes sense, as for those who are already socially isolated and have fewer opportunities to engage with their peers, school is an essential link to the community.

To conclude, these undertakings do not require enormous resource allocation to begin with.

Now and on the return to in-person learning, social supports are likely to make an important contribution to the learning of all students, but particularly those with disability.

## Supplementary Information

Below is the link to the electronic supplementary material.

Supplementary file1 (DOCX 24 kb)

## Data Availability

Data are propriety of Children and Young People with Disability Australia.

## Code Availability

Data analyzed using Stata software. Code available from the corresponding author upon request.
