# Supplementary material for: Successful School Interventions for Students with Disability During Covid-19: Empirical Evidence from Australia
Source: Asia-Pacific Edu Res. 2022 Apr 9;32(3):367–77. doi: 10.1007/s40299-022-00659-0 (PMC8994099; doi:10.1007/s40299-022-00659-0)
Supplement: Supplementary file 1 — Supplementary file1 (DOCX 24 kb) [file 40299_2022_659_MOESM1_ESM.docx]

**SUPPLEMENTARY MATERIAL: Technical Appendix for**

**Successful school interventions for students with disability during Covid-19: empirical evidence from Australia**

Australian Educational Researcher

A Publication of the Australian Association for Research in Education

Catherine Smith*

Melbourne Graduate School of Education, University of Melbourne, Melbourne, VIC

Massimiliano Tani

School of Business, University of New South Wales, Canberra, ACT

Sophie Yates

School of Business, University of New South Wales, Canberra, ACT

Helen Dickinson

School of Business, University of New South Wales, Canberra, ACT

* corresponding author:

Tel: + 61 0439 987 982

Email: catherine.smith1@unimelb.edu.au

*Empirical specification*

The regression analysis performed is based an Ordinary Least Squares (OLS) applied to the statistical model:

$$y_{i}=\alpha+X_{i}\beta+\gamma Z_{i}+\varepsilon_{i}$$

where:

$y_{i}$ is the educational outcome of interest experienced by student *i*. For example, in the case of whether the student receives adequate support in their education, the variable $y_{i}$ includes the values reported in the survey: namely, 5 if the survey respondent strongly agrees with the statement, 4 if s/he agrees, 3 for a neutral answer, 2 for disagreeing and 1 if the respondent strongly disagrees with the statement. A similar approach is used for each of the remaining outcomes summarised in 2-4 above, and run separate regressions for each of the four possible educational outcomes, generating four sets of results;

$X_{i}$ is a set of independent variables that control for gender, age group, support received before the pandemic, whether studying full-time, mental health status, non-English-speaking background, whether aboriginal or Torres Strait islander, if funded by NDIS, whether has individual education program in place, type of school, and location (urban and in which state);

$Z_{i}$ is the key explanatory variable, namely a set of answers about the support received during the pandemic. This set contains five components: namely, whether the support took the form of curriculum support and a support worker, specific aides and equipment, supervision, social support, and care services (assistance with personal care + behavioural support + access to specialist allied health).

We apply vector $Z_{i}$ in two alternative specifications: first, as a 3-category variable, which we label “version A” in the paper, with values of 0 if no support was received, 1 if only one type of support was received; and 2 if two or more types of support were received. In the second specification, we use the five components of vector $Z_{i}$ independently, as five separate indicators (“version B”).

**Are interventions effective?**

Table A1 reports the estimates obtained when using version A. The columns report the educational outcomes ($y_{i}$) while the rows report the control variables ${Z_{i},X}_{i}$ (the ‘slope’ of the regression line linking key explanatory variable and outcome) and a constant term (the ‘intercept’ of such relationship).

**Table A1: Regression results: educational outcomes**

| **Dependent variable 🡪**  ***Controls*** | **The student receives adequate support** | **The student is made to feel part of the learning community** | **The student is engaged in his/her learning** | **The student feels more socially isolated** |
| --- | --- | --- | --- | --- |
| ***Version A;***  ***1 type of intervention only*** | **.358*****  **(.109)** | **.240****  **(.112)** | **.105**  **(.117)** | **-.105**  **(.110)** |
| ***2+ types of intervention*** | **1.09*****  **(.115)** | **.881*****  **(.120)** | **.474*****  **(.130)** | **-.182***  **(.106)** |
|  |  |  |  |  |
| *Mental health* | -.412***  (.094) | -.297***  (.099) | -.487***  (.107) | .524***  (.093) |
| *School non-gov.t* | .318***  (.120) | .616***  (.119) | .297**  (.137) | -.078  (.116) |
| *School Other* | .258  (.174) | .265  (.174) | .371**  (.173) | .054  (.151) |
| *Student is full-time* | .135  (.175) | .127  (.186) | .035  (.198) | .540***  (.170) |
| *Student has no IEP* | .023  (.109) | .044  (.110) | -.169  (.118) | -.180*  (.103) |
| *Index Covid impact* | -.048*  (.025) | -.104***  (.024) | -.035  (.026) | .056***  (.021) |
| *Index support before Covid* | -.026  (.020) | .0000  (.021) | -.001  (.022) | .044**  (.021) |
| *Respondent is child* | .661**  (.267) | .405  (.253) | .675**  (.282) | .222  (.238) |
| *Gender: Other* | .412  (.420) | .322  (.357) | .282***  (.106) | -.075  (.271) |
| *Atsi* | .303  (.266) | .434*  (.252) | -.092  (.222) | .280  (.242) |
| *Metro area* | .040  (.099) | .162  (.099) | .143  (.104) | -.009  (.097) |
| *State fixed effects* | Yes | Yes | Yes | Yes |
| *Constant* | 2.11***  (.234) | 2.67***  (.243) | 2.87***  (.256) | 2.92***  (.234) |
| Variance explained | .2193 | .2259 | .1515 | .1232 |
| N | 618 | 616 | 615 | 616 |

Notes: Standard error in parentheses. Point estimates different from zero at 10%, 5%, and 1% level of statistical significance are starred with *, **, and ***.

*Effect of interventions*

As presented in the main text, the estimates support the hypothesis that the intervention has been both successful and very helpful for students with disabilities – the point estimates are indeed quite large. They are also positive for positive outcomes (implying they help to achieve them) and negative for the negative outcome (implying they contribute to reduce undesirable outcomes). Children and young people receiving support during the pandemic experienced a substantive and positive contribution in maintaining their learning engagement and reducing feelings of social isolation.

*Effect of other control variables*

The Index of support received during Covid, or the level of support received during the pandemic (version A), it is worth noting that Covid-19 had a large negative effect on mental health, and this is perhaps the largest effect in terms of size and statistical significance of the point estimate: about 30-50% of respondents admit to have been seriously affected by Covid-19 in their levels of stress and anxiety. The strong association between mental health and learning outcomes highlights the importance that emotional conditions have on learning activity, and the fact that Covid-19 hit the most on the stability of emotional status.

Another important determinant is the type of school attended: non-government schools appear to be more active in providing support, but this result could be entirely driven by unobserved factors, such as the resources available to the school, that cannot be better controlled with the cross-sectional data at hand.

**Which intervention was most effective?**

When the ‘Index during Covid’ variable uses version B, the effect of the various components is separately estimated (Table A2). These results can be directly compared with the previous results. Most coefficients and standard errors are in fact identical or very similar aside from the components of the version B displayed below.

**Table A2: Regression extension on what support worked: educational outcomes**

| **Dependent variable 🡪**  ***Controls*** | **The student receives adequate support** | **The student is made to feel part of the learning community** | **The student is engaged in his/her learning** | **The student feels more socially isolated** |
| --- | --- | --- | --- | --- |
| ***Index support during Covid (version B):*** | | | | |
| ***Education support*** | **.447*****  **(.096)** | **.291*****  **(.100)** | **.074**  **(.106)** | **-.009**  **(.093)** |
| ***Specific aides and equipment*** | **.489*****  **(.164)** | **.328****  **(.154)** | **.108**  **(.172)** | **-.257***  **(.140)** |
| ***Supervision*** | **.380****  **(.164)** | **.421****  **(.164)** | **.315***  **(.180)** | **.104**  **(.138)** |
| ***Social support*** | **.525*****  **(.189)** | **.440****  **(.182)** | **.575*****  **(.197)** | **-.308****  **(.160)** |
| ***Care services*** | **.163**  **(.136)** | **.172**  **(.128)** | **.029**  **(.143)** | **-.107**  **(.119)** |
|  |  |  |  |  |
| *Mental health* | -.408***  (.094) | -.287***  (.098) | -.469***  (.106) | .512***  (.093) |
| *School non-gov.t* | .328***  (.116) | .628***  (.117) | .307**  (.134) | -.088  (.119) |
| *School Other* | .249  (.172) | .239  (.176) | .336*  (.177) | .072  (.153) |
| *Student is full-time* | .118  (.173) | .110  (.185) | .012  (.198) | .549***  (.171) |
| *Student has no IEP* | .081  (.110) | .097  (.108) | -.134  (.117) | -.167  (.102) |
| *Index Covid impact* | -.045*  (.025) | -.102***  (.024) | -.032  (.026) | .054**  (.021) |
| *Index support before Covid* | -.034  (.020) | -.006  (.021) | -.004  (.022) | .049**  (.021) |
| *Respondent is child* | .474*  (.269) | .239  (.258) | .607**  (.296) | .289  (.238) |
| *Gender: Other* | .286  (.447) | .229  (.379) | .275***  (.105) | -.005  (.271) |
| *Atsi* | .333  (.256) | .456*  (.243) | -.084  (.214) | .260  (.240) |
| *Metro area* | .070  (.096) | .195**  (.098) | .174*  (.101) | -.008  (.096) |
| *State fixed effects* | Yes | Yes | Yes | Yes |
| *Constant* | 2.11***  (.231) | 2.68***  (.242) | 2.89***  (.254) | 2.87***  (.232) |
| Variance explained | .2374 | .2366 | .1693 | .1358 |
| N | 618 | 616 | 615 | 616 |

Notes: Standard error in parentheses. Point estimates different from zero at 10%, 5%, and 1% level of statistical significance are starred with *, **, and ***.
